# The Enhancement of Enargite Dissolution by Sodium Hypochlorite in Ammoniacal Solutions

**DOI:** 10.3390/ma14164529

**Published:** 2021-08-12

**Authors:** Lilian Velásquez-Yévenes, Hans Álvarez, Víctor Quezada, Antonio García

**Affiliations:** 1 Escuela de Ingeniería Civil de Minas, Facultad de Ingeniería, Universidad de Talca, Curicó 3340000, Chile; 2 Laboratorio de Investigación de Minerales Sulfurados, Departamento de Ingeniería Metalúrgica y Minas, Universidad Católica del Norte, Avenida Angamos 0610, Antofagasta 1270709, Chile; haahans@gmail.com (H.Á.); vquezada@ucn.cl (V.Q.); agarcia@ucn.cl (A.G.)

**Keywords:** enargite dissolution, ammonia salts, sodium hypochlorite, pretreatment with NaCl, curing time

## Abstract

The dissolution of both copper and arsenic from a copper concentrate was investigated in oxidative ammonia/ammonium solutions at moderate temperatures and atmospheric pressure. The main parameters studied were temperature, pH, concentrations of different ammonia salts, the presence of sodium hypochlorite, pretreatment with sodium chloride, and curing period. In all ammoniacal solutions studied, increasing the temperature enhanced the dissolution of copper, but the dissolution of arsenic remained marginal. Mixing the copper concentrate with sodium chloride and leaving it to rest for 72 h before leaching in ammoniacal solutions significantly increased the dissolution of copper and slightly increased the dissolution of arsenic from the concentrate. A maximum of 35% of Cu and 3.3% of As were extracted when ammonium carbonate was used as the lixiviant. The results show relatively rapid dissolution of the concentrate with the addition of sodium hypochlorite in ammonium carbonate solution, achieving a dissolution of up to 50% and 25% of copper and arsenic, respectively. A copper dissolution with a non-linear regression model was proposed, considering the effect of NaClO and NH_4_Cl at 25 °C. These findings highlight the importance of using the correct anionic ligands for the ammonium ions and temperature to obtain a high dissolution of copper or arsenic. The results also showed that the curing time of the packed bed before the commencement of leaching appeared to be an important parameter to enhance the dissolution of copper and leave the arsenic in the residues.

## 1. Introduction

According to [1], more than 300 arsenic (As) minerals are known to occur in nature. Of these, approximately 60% are arsenates, 20% are sulfides and sulfosalts, 10% are oxides, and the rest are arsenite, arsenide, native elements, and metal alloys. The most important primary As-bearing minerals are those where the arsenic occurs as either the anion (arsenide) or di-anion (diarsenide), or as the sulfarsenide-anion; these anions are bonded to metals such as Fe (arsenopyrite), Co (cobaltite), Ni (gersdorffite), and Cu (enargite, tennantite). Arsenic is one of nature’s most toxic elements, and excessive arsenic exposure has been linked to skin lesions, increased bladder, and lung cancer mortality in Northern Chile [2].

One of the most dreaded environmental impacts is related to the release of arsenic into the environment. Ores and concentrates from flotation containing high concentrations of arsenic are considered a potential hazard, requiring special precautions in the beneficiation and smelting processes. According to [3], the annual copper output from ore smelting reaches more than 7 million tons in China. Generally, the contents of As and Cu in copper concentrate are 0.2% and 25%, respectively. However, the cumulative total arsenic brought into copper smelting systems is estimated to be more than 56,000 tons every year. According to [4], 10 tons of arsenic associated with copper concentrates are emitted each day from the seven copper smelting plants in Chile, the largest producer of copper in the world. Along with the smelting process of copper, arsenic is successively removed and transformed into various byproducts such as slags, flue dust, black copper, anode slime, etc. [3]. These Arsenic compounds contain byproducts that could be processed to either recover the valuable metals or be discarded as waste in dumps. Due to the small market capacity of arsenic-related products, arsenic has little commercial value to be further exploited. Hence, seeking efficient and safe ways for the disposal of arsenic is always of great significance.

The presence of copper deposits with arsenic content in the Antofagasta Region of Chile is widely known [5,6,7,8]. The arsenic-bearing copper minerals such as enargite (Cu_3_AsS_4_) and tennantite (Cu_12_AsS_13_) attract considerable interests because of their high copper content [9]. However, the presence of arsenic in the copper concentrate is a challenge due to its subsequent content in the metallic copper. Because of the toxicity of arsenic, the smelting industry is forced to be selective in the type of copper concentrates used. By regulation, concentrates can have a maximum content of 0.5% arsenic [10], but this is expected to be reduced shortly. Hence, there is a great need for finding alternative, cleaner, and effective treatments for arsenide copper minerals. A roasting technique was commonly used to eliminate most of the arsenic, but nowadays, this process requires alternatives due to the stringent environmental regulations [11]. The hydrometallurgical process seems to be a cleaner route to process complex ores such as enargite. However, there was a crucial problem with developing a leach process for copper sulfide minerals as the leaching is inhibited at ambient temperature. Several studies reported the formation of a layer on the surface of the sulfide minerals during dissolution. Some researchers claim that this layer could be composed of elemental sulfur or a copper-rich polysulfide layer. However, the nature of this “passivating layer” has not yet been established with certainty. The layer inhibits contact between the mineral and the oxidizing agents, which reduces the dissolution rate [12,13,14,15,16,17,18]. Over the years, research has demonstrated some success in adding chloride [14,15,16,17,18,19,20,21,22] and nitrate to the acid leaching solution [23]. However, in the treatment of ores with high consumption of acid, these processes become costly. Therefore, the new option requires the use of an alkaline leaching solution.

The alkaline solutions that can be used to dissolve copper from enargite are cyanide and ammonia. However, the use of cyanide is not preferable due to its instability, toxicity, and complications in the recovery of copper from cyanide solutions. In ammonia media, Cu^2+^ ions form stable ammine complexes that could be extracted by the conventional solvent extraction process (copper leaching in acid media). The essential advantage of leaching in an alkaline media such as ammonium salts is the ability to recover the dissolved metal by direct electrodeposition or by precipitation with sulfur compounds. As ammonia can only exist in a media of moderate alkalinity (pH = 8.7–9.8), the obtained PLS contains very few impurities, which facilitates its subsequent purification, making it more straightforward [24].

The first report using ammoniacal leaching on an industrial scale was in Kennecott, Alaska, where copper ore was present in a matrix containing carbonates (limestone-dolomite) [25,26]. Another process described as one of the first industrial-scale processes corresponds to the method developed by Sherritt-Gordon in a plant in Fort Saskatchewan, Canada, in 1953. In this process, the ammoniacal leaching of sulphate ores containing Cu, Ni and Co was carried out in autoclaves at high pressures and temperatures using oxygen as an oxidizing agent [27]. Other processes were the Arbiter process for recovering copper from chalcopyrite concentrates in the presence of oxygen [28] and the Escondida process to produce high-grade copper concentrates [29].

On the other hand, hypochlorite ion (OCl^−^), a strong oxidizing agent, has been used as an alternative method to remove arsenic from copper concentrates [30,31]. The topochemical reaction occurs according to Reaction (1). Arsenic dissolves into the solution, and copper stays as a CuO in the residue. In the second stage, the copper residue is leached.

(R1) $${\mathrm{Cu}}_{2}{\mathrm{AsS}}_{4}+11{\mathrm{OH}}^{-}+17.5{\mathrm{ClO}}^{-}\;\to \;3\mathrm{CuO}+\mathrm{As}O_{4}^{3-}+4SO_{4}^{2-}+5.5H_{2}O+17.5{\mathrm{Cl}}^{-}$$

In order to minimize the problems associated with the arsenic content in copper concentrate, this paper presents results from a study on the leaching of enargite in ammoniacal solutions at various alkaline pH, anionics ligands, chloride ions, using a strong oxidizing (OCl^−^), variation of temperature and curing time.

## 2. Experimental

### 2.1. Copper Concentrate Sample

The copper concentrate sample was obtained from a flotation plant. The sample was prepared to a size fraction of −38 μm. The chemical composition of the screened sample was determined using inductively coupled plasma atomic emission spectroscopy (ICP-AES) (Optima 2000 DV, PerkinElmer, Überlinge, Germany). The chemical composition of the sample was 36.2% of Cu, 14.9% of Fe and 4.3% of As (Table 1). Mineralogical data were obtained by QEMSCAN, using a Model Zeiss EVO 50 (Zeiss, Oberkochen, Germany), with Bruker AXS XFlash 4010 detectors (Bruker, Billerica, MA, USA) and Software iDiscover 5.3.2.501 (FEI Company, Brisbane, Australia). For the QEMSCAN analysis performed, the Bulk Mineral Analysis (BMA) technique was used. In a BMA, the analysis points are distributed over the entire surface of the briquette, according to a previously established collection grid. In the “X” coordinates a “pixel spacing” is established, while in the “Y” coordinate the term “line spacing” is used. BMA reported a robust quantification of modal mineralogy (total ore), allowing at the same time to obtain the contribution (main element) that each mineral species present. The major minerals identified were pyrite, enargite, chalcocite, chalcopyrite and covellite (Table 2). According to Table 2, enargite is the principal copper mineral in the sample.

### 2.2. Leaching in Shake Flasks

Leaching experiments of enargite concentrate were conducted in 250 mL shake flasks at 25 °Cand 35 °C, with stirring speed of 140 rev/min for 8 h. 10 g of copper concentrate sample was used for all leaching tests. Leach solutions were prepared in a volume of 500 mL using distilled water. Three different salts were investigated, NH_4_Cl, (NH_4_)_2_SO_4_ and (NH_4_)_2_CO_3_, in a concentration of 1.5 M. To study the effect of pretreatment in the dissolution of Cu and As, selected tests were performed by mixing the copper concentrate with 15 g/L of NaCl and left to rest for 24 or 72 h before leaching. All leach solutions contained 0.5 g/L of cupric ions, which is the typical concentration in a raffinate in any Chilean plant. The effect of the addition of NaClO complexing and oxidizing agent was also investigated by adding 10 g/L to the leaching solution in ammonia media. Table 3 shows a summary of the shake flasks leaching tests conditions. Finally, the modelling of copper extraction with non-linear regression was studied using Minitab 18 computational tool (Minitab LLC, State College, PA, USA), considering the effect of NaClO and NH_4_Cl at 25 °C.

## 3. Results and Discussions

**Figure 1:**
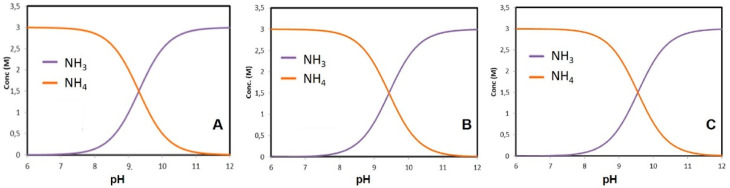
Distribution diagram of NH_3_/NH_4_^+^ from varying the NH_3_–NH_4_^+^ concentration of the solution in the presence of different ammonium salts, NH_4_Cl (**A**), (NH_4_)_2_SO_4_ (**B**) and (NH_4_)_2_CO_3_ (**C**).

Based on the study by [32], species distribution diagrams were constructed for the different ammonium salts and working conditions used in this research. Figure 1 shows the pKa values for ammonium chloride, ammonium carbonate and ammonium sulfate. It can be seen that as the pH increases, the ammonia concentrations increase, while the ammonium concentrations decrease. This is due to the transformation that occurs from ammonium to ammonia. However, a point was reached where both curves converge; this is an equilibrium point where the concentrations of ammonium and ammonia are equal. This so-called pKa must be the desired and optimal concentration to obtain the best dissolutions. The pKa of ammonium chloride was reported to be 9.50 [33], which is consistent with the results shown on Figure 1. According to Figure 1, pKa values of 9.50, 9.43 and 9.55 were obtained in solutions contained NH_4_Cl, (NH_4_)_2_CO_3_ and (NH_4_)_2_SO_4_, respectively. Therefore, the study will be conducted by using 1.5 M of each ammonium salts.

### 3.1. Effect of Temperature on the Dissolution of Copper and Arsenic

**Figure 2:**
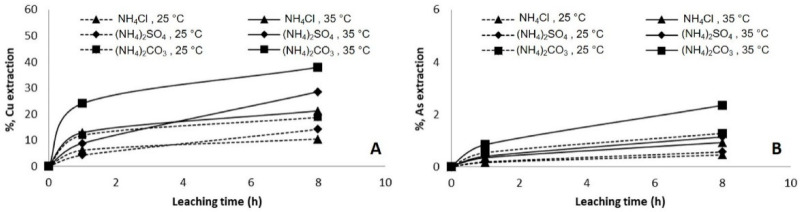
Copper (**A**) and arsenic (**B**) extraction in different ammonia solution at 25 °C and 35 °C.

Figure 2 shows the results of Cu and As extraction from copper concentrate in shake flasks at varying temperature and ammonia salts. Leaching tests were performed with 1.5 M of three different ammonia salts (NH_4_Cl, (NH_4_)_2_SO_4_ and (NH_4_)_2_CO_3_) at 25 °C and 35 °C. Increasing the temperature resulted in an increase in both copper and arsenic extraction. The highest dissolution of copper of up to 40% was achieved when (NH_4_)_2_CO_3_ was used, followed by (NH_4_)_2_SO_4_ (30%) and NH_4_Cl (20%), all of them at 35 °C. At 25 °C, the three different tests achieved no more than 10% of copper dissolution. It is interesting to note that there was a limited dissolution in arsenic, less than 1% for all condition and temperature. A slight increase in arsenic dissolution was obtained when (NH_4_)_2_CO_3_ was added at 35 °C. It could be inferred that enargite dissolved at a lower rate than the other copper minerals present in the concentrate (Table 2).

### 3.2. Effect of Pretreatment and Curing Time

**Figure 3:**
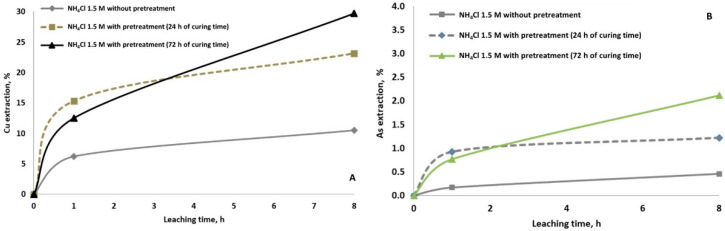
Copper (**A**) and arsenic (**B**) extraction at ambition condition using 1.5 M of NH_4_Cl without and with pretreatment (15 g/L of NaCl and curing time of 24 h and 72 h).

From Figure 3, it is evident that the copper (Figure 3A) and arsenic (Figure 3B) dissolutions were enhanced by the pretreatment with NaCl and the rest period of 24 h before leaching with NH_4_Cl solution. The possible chemical reaction is shown in Reaction 2. Still, also it is suggested that during the curing time, enargite can be decomposed to Cu_2_S or CuS and then dissolved with the irrigation of ammonia solution. The dissolution of copper achieved was 23% with pretreatment versus 10% without pretreatment; and the dissolution of arsenic increased slightly from 0.5% to 1%. According to [34], curing time generates a homogeneous distribution of the mineral bed’s leaching solution, and hence increasing the Cu dissolution. It was observed that the longer the curing time, the higher the copper and arsenic extraction. Curing time for 72 h resulted in 30% final copper extraction, which is 6.6% increase compared to 24 h. The use of chloride/ammonia has an additional advantage. On the one hand, it fulfils the objective, just as other chlorides do, of providing the solution with the appropriate concentration of chloride ion. Ammonia dissolved in water forms a strongly alkaline solution of ammonium hydroxide. The high solubility of ammonia in water is the result of the tendency of the two to interact with each other through a hydrogen bond (Reaction 3).

2Cu_3_AsS_4_ + 6H_2_SO_4_ + 5.5O_2_ → 6CuSO_4_ + 2H_3_AsO_4_ + 8S° + 3H_2_O (R2)

(R3) $$\mathrm{NH}3_{\left(\mathrm{aq}\right)}+H^{+}\;\to \;NH_{4}^{+}$$

**Figure 4:**
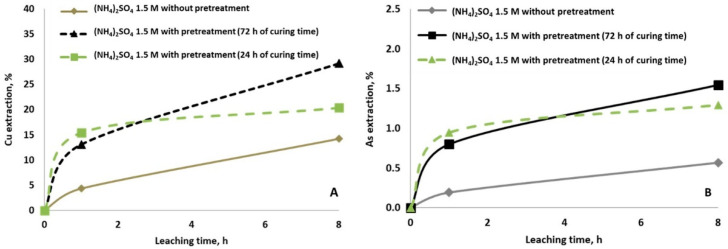
Copper (**A**) and arsenic (**B**) extraction at ambient condition using 1.5 M of (NH_4_)_2_SO_4_ without and with pretreatment (15 g/L of NaCl and curing time of 24 h and 72 h).

Similar results were obtained when the copper concentrate was mixed with NaCl and left to rest for 24 h or 72 h before leaching with (NH_4_)_2_SO_4_. The dissolution of copper and arsenic increased with the pretreatment but at a lower rate (Figure 4). Increasing the curing period to 72 h resulted in an increase in the dissolution of copper (Figure 4A) and arsenic (Figure 4B), reaching an extraction of 30% and 1.5% after 8 h of leaching, respectively.

**Figure 5:**
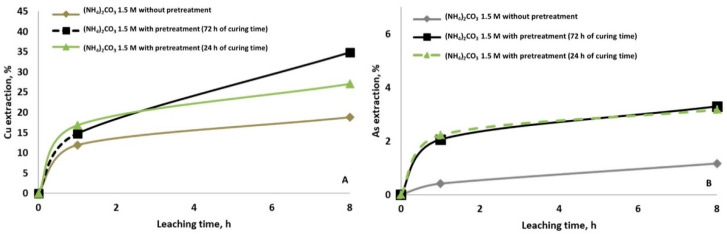
Copper (**A**) and arsenic (**B**) extraction at ambient condition using 1.5 M of (NH_4_)_2_CO_3_ without and with pretreatment (15 g/L of NaCl and curing time of 24 h and 72 h).

Tests performed with the (NH_4_)_2_CO_3_ media are shown in Figure 5. As expected, the highest dissolution was obtained in this media, 27% of copper and 3.0% of arsenic, when the concentrate was mixed with NaCl and left to rest for 24 h. When the curing period was increased to 72 h, an increase was observed in the extraction of copper around 35%, however, the arsenic extraction remained the same as the 24 h curing time pretreatment. It is inferred that the other copper minerals contained in the concentrate might dissolve rather than the enargite. A long curing period before the leaching stage was identified as favourable for the dissolution of sulphide ores such as chalcopyrite [21,35] or chalcocite [36]. The advantage of using this media is the buffering effect of the alkaline ammonium carbonate solution, which makes the pH remain practically constant over time without the need for any adjustment.

### 3.3. Effect of NaClO

**Figure 6:**
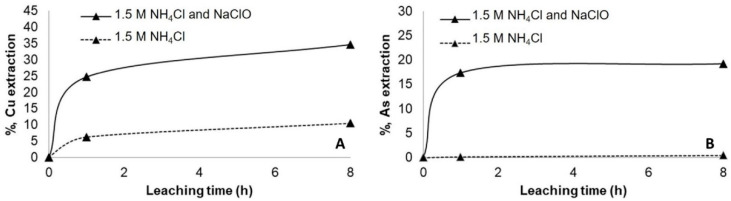
Copper (**A**) and arsenic (**B**) extraction at ambient condition using 1.5 M of (NH_4_)Cl with and without the addition of 10 g/L NaClO.

**Figure 7:**
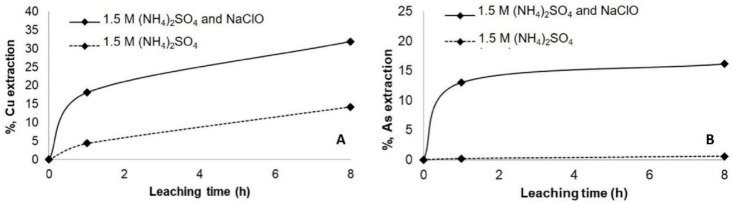
Copper (**A**) and arsenic (**B**) extraction at ambient condition using 1.5 M of (NH_4_)_2_SO_4_ with and without the addition of 10 g/L NaClO.

**Figure 8:**
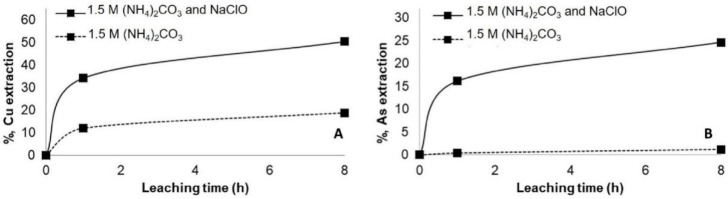
Copper (**A**) and arsenic (**B**) extraction at ambient condition using 1.5 M of (NH_4_)_2_CO_3_ with and without the addition of 10 g/L NaClO.

The results indicate that copper extraction increased by around 35% (Figure 6). Still, this result is comparable with the result obtained when the leaching was carried out in NH_4_Cl after the pretreatment with NaCl and 72 h of curing period. Nevertheless, a considerable increase in arsenic dissolution was observed by almost 20%, agreed with the result of [31] (Figure 6B). The passivation of enargite in this media was evident after 2 h of leaching. The same effect was observed when (NH_4_)_2_SO_4_ was used with NaClO (Figure 7). Due to acid generations, monitoring of oxidizing pH conditions and potential reduction is essential to continue the effectiveness of the leaching test [37]. As it was expected, the leaching of the concentrate with (NH_4_)_2_CO_3_ and NaClO achieved the highest dissolution of copper and arsenic, around 50% and 25% of copper and arsenic, respectively (Figure 8). These results highlight the importance of using an oxidant such as NaClO if the objective is to remove arsenic from the concentrate [38].

### 3.4. Modelling of Copper Extraction in Function of NH_4_Cl, NaClO and Leaching Time

The addition of NaClO to the alkaline leaching solution enhanced the dissolution of copper at 25 °C. However, the major increase was observed when NH_4_Cl was used as the lixiviant. The dissolution of copper increased from 10% to 35% when NaClO was added.

For the modelling of the dependent variables on the copper dissolution, the support of statistical software was necessary since the experimental curves show strongly non-linear behaviour. In this investigation, the Minitab 18 computational tool was used and the results with copper extraction using NH_4_Cl and NaClO considered. After extensive model testing, the following (Equation (1)) was chosen due to its better fit:

% Cu dissolution = (a0 + a1 × [NaClO]) × (1 − b0 × e ^(−b1×leaching time)^) (1)

With coefficients a0, a1, b0 and b1, NaClO concentration and leaching time are measured in g/L and hours, respectively. The Levenberg-Marquardt algorithm was applied to the model for calculating its parameters or coefficients. Table 4 shows estimate, standard error (SE) and confidence interval (CI) of each coefficient of the model. The resulting equation is exposed in Equation (2).

% Cu dissolution = (9.96826 + 2.48720 × [NaClO]) × (1 − b0 × e^(−1.21448×leaching time)^) (2)

There is a 95% confidence interval containing the value of the parameter for the population. The parameter was statistically significant if the range excludes the value of the null hypothesis (the term containing the parameter without effect). For reference, in the case of linear regression, the null hypothesis value for each parameter was 0, so there is no effect. Table 5 exhibits a summary of the statistical parameters of the model fit.

**Figure 9:**
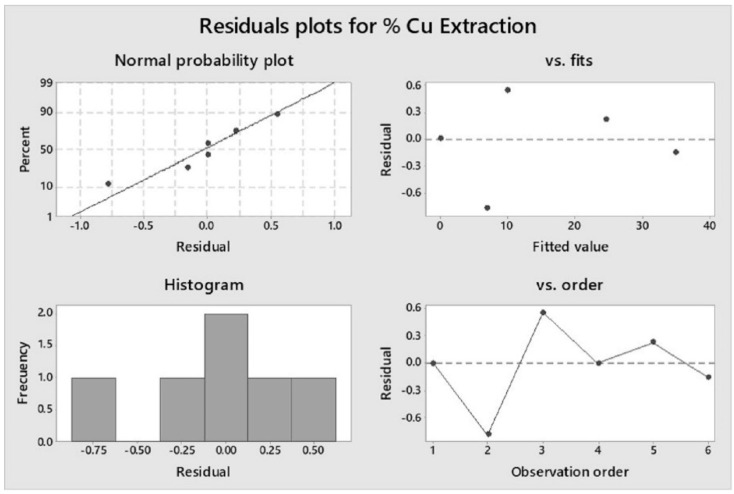
Residual plots for copper dissolution.

Figure 9 shows that in the plot of residuals vs. fits, it is verified that the residuals are randomly distributed and have a limited variance. The points were located randomly on both sides of 0. The plot of residuals vs. order shows that the residuals were independent of each other. The residuals show no trends or patterns when displayed in chronological order. From the normal probability plot of the residuals, it is verified that the residuals are normally distributed. The normal probability plot of the residuals follows approximately a straight line.

**Figure 10:**
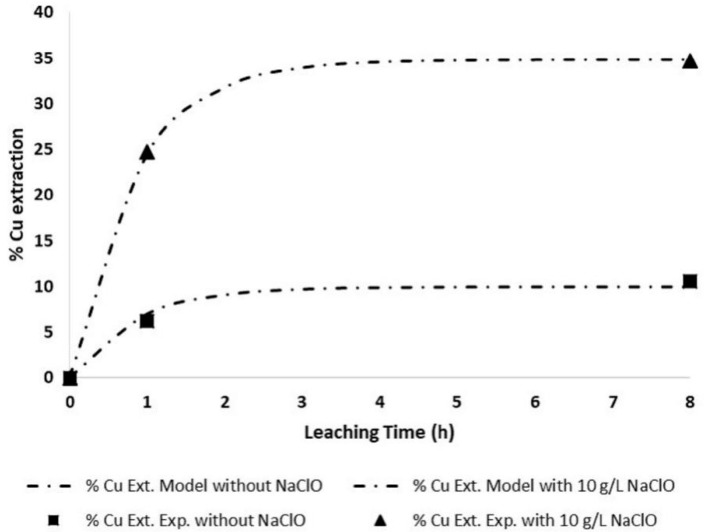
Experimental and modeled (• ▬ •) copper dissolution v/s time. Experimental test without NaClO and 1.5 M NH_4_Cl (■), and experimental test with 10 g/L NaClO and 1.5 M NH_4_Cl (▲).

For the maximum measured value of copper extraction, 34.68%, the relative error was 2.02%. Therefore, modelled curves of the copper dissolution reproduce well the behaviour of the experimental. The mathematical model fits well with the experimental data, as seen in Figure 10.

## 4. Conclusions

A comparative study of copper and arsenic dissolution from a copper concentrate using different ammonia salts under the ambient condition with the addition of chloride ions as pretreatment and hypochlorite as an oxidizing source was presented. Based on the results obtained, it can be concluded that:

- In all ammoniacal solutions studied, increased temperature enhanced the dissolution of copper, but no significant increase in the dissolution of arsenic was observed. Ammonium carbonate solutions at 35 °C dissolved the highest copper and arsenic amount.
- Mixing the copper concentrate with NaCl and leaving it to rest for 72 h before the leaching with ammoniacal solutions significantly increases the dissolution of both copper and arsenic from the concentrate. A maximum of 35% of Cu and 3.3% of As were extracted when ammonium carbonate was used as the lixiviant.
- The addition of an oxidizing agent such as hypochlorite ion, OCl^−^ to the alkaline leaching solution dramatically enhanced the dissolution of enargite contained in the concentrate obtained more than 50% of copper and 25% of As.
- With an error of 2.02%, Table 4 shows that the experimental results were in good agreement with the modelled equation (Equation (2)). Therefore, the mathematical model fits well with the experimental data.
- Although the results described above indicate that the leaching of enargite using ammonia solutions could be attractive from a kinetic point of view, many important practical considerations must be considered. As the cost of the lixiviants, a neutralizing reagent to maintain the pH at a level appropriate for efficient leaching.

## Figures and Tables

**Table 1:** Chemical composition of copper concentrates determined by XPS analysis.

Cu (%)	Cu_soluble_ (%)	Fe (%)	Mo (ppm)	Mo_soluble_ (ppm)	As (%)	S (%)
36.2	1.55	14.9	230	12.5	4.26	31.5

**Table 2:** Mineralogical composition of copper concentrate determined by Qemscan analysis.

Mineral	Mass (%)
Enargite	21.0
Pyrite	29.8
Chalcocite	17.9
Other Cu-minerals	7.79
Chalcopyrite	6.30
Covellite	4.58
Gangue	3.14
Bornite	2.92
Quartz	2.69
Tenantite	1.84
Sphalerite	1.42
Plagioclase	0.32
Feldspars	0.22
Mn-minerals	0.07
Molybdenite	0.05
As-minerals	0.01

**Table 3:** Shake flask leaching conditions of enargite concentrate.

Test	Temp. °C	Curing (h)	NH_4_Cl (M)	(NH_4_)_2_CO_3_ (M)	(NH_4_)_2_SO_4_ (M)	NaCl (g/L)	OCl^−^ (g/L)
1	25	-	1.5	-	-	-	-
2	35	-	1.5	-	-	-	-
3	25	-	-	1.5	-	-	-
4	35	-	-	1.5	-	-	-
5	25	-	-	-	1.5	-	-
6	35	-	-	-	1.5	-	-
7	25	24.0	1.5	-	-	15	-
8	25	24.0	-	1.5	-	15	-
9	25	24.0	-	-	1.5	15	-
10	25	72.0	1.5	-	-	15	-
11	25	72.0	-	1.5	-	15	-
12	25	72.0	-	-	1.5	15	-
13	25	-	1.5	-	-	-	10
14	25	-	-	1.5	-	-	10
15	25	-	-	-	1.5	-	10

**Table 4:** Parameters calculations associated with Cu dissolution model.

Parameter	Estimate	SE of Estimate	CI of 95%
a0	9.96826	0.585156	(7.46087;12.5206)
a1	2.48720	0.085869	(2.12372;2.8583)
b0	1.00000	0.019377	(0.91634;1.0837)
b1	1.21448	0.082207	(0.91619;1.6699)

**Table 5:** Statistical summary of the fit model.

Parameter	Value	Meaning
SSE final	0.986153	Sum of squared residual
DFE	2.000000	Degrees of freedom for error, are equal to the sample size, plus 1
MSE	0.493077	Means square of the error, is the variance around the fitted values. MSE = SSE/DFE
S	0.702194	Distance (of error) between the data values and the fitted values. S = MSE ^1/2^

## Data Availability

Data sharing is not applicable.
